# Neuropathic Pain Relief Through Transcutaneous Electrical Neuromuscular Stimulation: Insights From a Systematic Review and Meta‐Analysis of Clinical Evidence

**DOI:** 10.1155/bmri/5328365

**Published:** 2025-10-22

**Authors:** Mohamed Magdy ElMeligie, Yasmine Sabry Gomaa, Ebtehal Taha, Efrem Kentiba, Heba Ahmed Ali Abdeen, Dina Mohamed Ali Al-Hamaky, Doaa Ibrahim Amin

**Affiliations:** ^1^ Department of Basic Sciences for Physical Therapy, Faculty of Physical Therapy, Al-Hayah University, Cairo, Egypt; ^2^ Department of Physical Therapy for Neuromuscular Disorders, Faculty of Physical Therapy, Kafr Elsheikh University, Kafr El Sheikh, Egypt; ^3^ Department of Physical Therapy for Neuromuscular Disorders, Faculty of Physical Therapy, Ahram Canadian University, 6th of October, Egypt, acu.edu.eg; ^4^ Department of Physical Therapy for Paediatrics and Women Health, Faculty of Physical Therapy, Ahram Canadian University, 6th of October, Egypt, acu.edu.eg; ^5^ Department of Sports Science, College of Natural and Computational Sciences, Arba Minch University, Arba Minch, Ethiopia, amu.edu.et; ^6^ Department of Physical Therapy for Cardiovascular, Respiratory Disorders and Geriatrics, Faculty of Physical Therapy, Cairo University, Giza, Egypt, cu.edu.eg; ^7^ Department of Physical Therapy for Musculoskeletal Disorders and Its Surgery, Faculty of Physical Therapy, Cairo University, Giza District, Egypt, cu.edu.eg; ^8^ Department of Physical Therapy for Basic Sciences, Faculty of Physical Therapy, Cairo University, Giza, Egypt, cu.edu.eg

**Keywords:** electric stimulation therapy, neuropathic pain, neuropathy, TENS, transcutaneous electrical nerve stimulation

## Abstract

**Objective:**

Transcutaneous electrical neuromuscular stimulation (TENS) is a noninvasive, nonpharmacological, and least expensive technique to improve sensitivity and reduce pain in patients with neuropathy. Evidence reported on the effectiveness of TENS on neuropathy was inconclusive. This study was aimed at providing recent evidence on the effectiveness of TENS for neuropathic pain, using data from randomized controlled trials and nonrandomized studies.

**Methods:**

We conducted a comprehensive search across Scopus, CINAHL, Web of Science, Medline, Embase, Cochrane Central Register of Control Trials (CENTRAL), Physiotherapy Evidence Database (Pedro), and Google Scholar from January 1, 1999, to May 5, 2024. Eligible studies included randomized controlled trials and nonrandomized studies on TENS for neuropathic pain, without restrictions based on language or country. Key inclusion criteria were studies on TENS for neuropathic pain, while exclusion criteria included studies on neuropathic pain due to cancer, HIV infection, or stroke. Two independent reviewers selected studies, extracted data, and assessed quality using the GRADE tool.

**Results:**

Thirty articles met the eligibility criteria, and 25 were included in the meta‐analysis. Overall, TENS slightly reduced neuropathic pain compared to placebo, but this was not clinically significant (SMD = −0.35; 95% CI: −0.90 to 0.10, *p* = 0.13). For patients with diabetic neuropathic pain, TENS was similar to placebo and other electrotherapies. However, it was better than placebo and other interventions for reducing neuropathic pain in spinal cord injury patients (SMD = −1.14; 95% CI: −2.22 to −0.06, *p* = 0.04).

**Conclusions:**

TENS generally seems to provide a small reduction in neuropathic pain compared with a placebo, other electrotherapies, or other interventions.

## 1. Introduction

Health problems due to chronic pain are escalating, particularly with the increase in life expectancy [1]. Neuropathic pain is chronic pain caused by somatosensory system disease or damage [2], resulting in burning and electrical‐like sensations and pain from nonpainful stimulations distributed according to the subjacent condition [3]. Patients with neuropathic pain may experience sleep disturbances, anxiety, depression, and a decreased quality of life [3]. Although different health conditions cause neuropathic pain, similar clinical manifestations are observed in these patients [4]. However, neuropathic pain is absent in all patients with peripheral neuropathy or central nervous system injury [5, 6].

Neuropathic pain is central or peripheral depending on the lesion or disease level [3, 7]. The prevalence of neuropathic pain in the general population ranges from 7% to 10% [8]. This condition is most common in women and patients over 50 years of age and mainly affects the lower back, lower limbs, neck, and upper limbs [9]. The prevalence and incidence of peripheral neuropathic pain may increase with the population’s aging [10].

The options for treating neuropathy associated with any illness are limited, especially for nonpharmacological treatments [4]. Treatment limitations may contribute to more than 50% of patients experiencing neuropathic pain failing to report or seek medical help [5]. Physicians usually prescribe treatments, such as anticonvulsants, analgesics, or antidepressants. The responses to these treatments vary significantly depending on the patient and the severity of neuropathy. Evidence regarding the effectiveness of the available interventions for neuropathic pain showed modest efficacy, with the risk of side effects that limit their use, making other interventions necessary [11]. According to numerous studies [12, 13], nonpharmacological treatments may reduce pain in patients experiencing neuropathic pain compared to pharmacological options, promoting a better quality of life. Electrical muscle stimulation is one of many benign therapies for neuropathic pain [14]. Transcutaneous electrical neuromuscular stimulation (TENS) is a safe and simple method of electrical stimulation. A previous systematic review [15] showed that TENS improved sensitivity and reduced pain in patients with neuropathy. However, that review included only a few trials with small sample sizes and low methodological quality. Therefore, the present review is aimed at generating recent evidence on the effectiveness of TENS for neuropathic pain from randomized controlled trials (RCTs) and nonrandomized studies (NRSs).

## 2. Materials and Methods

### 2.1. Literature Search

We used the Preferred Reporting Items for Systematic Reviews and Meta‐Analyses guidelines (PRISMA) to report the systematic review [16]. The completed PRISMA 2020 checklist details compliance with reporting guidelines for systematic reviews, including itemized responses to each PRISMA criterion (File S1). An independent researcher (D.A.) applied specific search strategies to the following online databases: CINAHL, Web of Science, Medline, Embase, Cochrane Central Register of Control Trials (CENTRAL), Physiotherapy Evidence Database (Pedro), and Google Scholar, from January 1999 to 5 May 2024. The search period was restricted to include only recent evidence. We only included articles published in peer‐reviewed journals and written in English. Data from patients presenting neuropathic pain due to cancer, HIV infection, or stroke were excluded.

A PICOT approach formulated the research question [17]. To develop the Medline search strategy, keywords (transcutaneous electric neurostimulation, TENS, neuropathic pain, randomized controlled trial) were combined. The remaining search strategies were adapted from the Medline search strategy.

### 2.2. Selection Criteria

After removing duplicates, two independent researchers (H.A. and D.A.) scrutinized the studies retrieved from the database searches to assess their eligibility by reading their titles, abstracts, and full texts. Consensus or consulting a third researcher (P.N.) resolved all disagreements or discrepancies.

### 2.3. Data Extraction

An independent researcher (P.N.) extracted information from the eligible articles using a preplanned Excel spreadsheet. The spreadsheet was developed to record the first author’s name, year of publication, sample size, type of intervention (i.e., TENS, placebo or sham treatment, drug treatment, or other physical therapy interventions), intervention characteristics (i.e., frequency, duration, and intensity), patient characteristics at baseline (i.e., mean age, sex, average pain, and symptom duration), health condition, pain measurement tool, and the trial duration.

### 2.4. Risk of Bias Assessment

Two independent researchers (M.E. and A.J.) used the PEDro scale to assess the risk of bias in each trial [18]. The PEDro scale is an 11‐item rating scale used to evaluate a clinical trial’s internal validity and determine whether it contains sufficient statistical information to make it interpretable. Because criterion one on the scale relates to external validity, it does not count in the final methodological quality. Thus, the maximum score on the PEDro scale is 10 points. The remaining criteria assess the internal validity (Criteria 2–9) and interpretability of the findings (Criteria 10 and 11). The internal validity criteria evaluate the risk of bias before the treatment phase, the performance bias during treatment, and the data analysis bias. The scale investigates the risk of bias related to the interpretability of the findings by examining between‐group statistical comparisons, reporting point estimates, and measures of variability. Any inconsistencies related to any aspect of the selected articles were resolved by consensus or by consulting a third researcher (P.N.).

### 2.5. Summary of Evidence

Two independent reviewers (M.E. and P.N.) assessed the overall quality of the evidence and the strength of the recommendation for pain reduction using the Grades of Recommendation, Assessment, Development, and Evaluation (GRADE) approach, as recommended in the Cochrane Handbook for Systematic Reviews of Interventions [19]. Based on the GRADE framework, we ranked the quality of the evidence as high, moderate, low, or very low. In addition, we used the study design, risk of bias, consistency of the results, directness of the results (generalizability of the findings), precision of the data to produce narrow confidence intervals (CIs), and other factors (e.g., reporting bias and publication bias) to decrease the quality of evidence.

According to the criteria presented above, the two researchers classified the evidence as high‐quality: at least 75% of eligible studies had consistent findings with a low risk of bias, direct and precise data, and no known or suspected publication bias. Further research is likely to maintain the same results. Moderate‐quality evidence: One of the GRADE domains was not met, and future research is expected to impact the result estimates. Low‐quality evidence: Two GRADE domains still need to be met, and future research will likely change the result estimates. Very low‐quality evidence: Three GRADE domains still required to be completed. Single studies with a sample size < 300 and a high risk of bias (PEDro score < 6) were considered to yield very low‐quality evidence or low‐quality evidence if there was a low risk of bias (PEDro score ≥ 6) [20]. In this case, the results are uncertain. Therefore, we excluded studies with a high risk of bias from the meta‐analysis.

### 2.6. Data Synthesis

Pain intensity scores are presented as mean differences (MDs) and 95% CIs. The results with a negative MD indicate that electrical muscle stimulation is more beneficial than the comparators. Our analysis included the outcome data from the last follow‐up period. We also determined whether the results were clinically relevant. A pain reduction of 2.3 points (0–10 scales) in the MD was considered to represent a minimally clinically significant change (MCIC) [21].

We used the chi^2^ and *I* ^2^ statistics to measure the heterogeneity between the eligible studies. Nonsignificant values in the chi‐square test (*p* > 0.05) and *I* ^2^ scores lower than 40% were considered unimportant [19]. Cases in which four or more studies compared the same intervention were pooled in a meta‐analysis using a random effects model. Subgroup analyses for each health condition were performed when possible. We used funnel plots to explore the possibility of publication bias [22]. Results that were not included in the meta‐analysis are presented descriptively. We used RevMan Version 5.3 to perform all data synthesis.

### 2.7. Patient and Public Involvement

We conducted no primary research involving patients because the systematic review focused on published literature.

## 3. Results

### 3.1. Study Selection

The initial search identified 6904 studies. After removing duplicates and screening titles and abstracts, 63 full texts were read. Finally, 30 articles with a total sample size of 1354 participants (mean = 54.1, range = 20–139) fulfilled the eligibility criteria. Twenty‐seven studies (90%) were RCTs, and three, or 10%, were conducted using designs other than RCT. Thirty studies were included in qualitative synthesis, and 25 studies (83%) were included in quantitative synthesis or meta‐analysis (Table 1).

**Table 1:** Characteristics of the included studies.

**Author**	**Intervention characteristics**	**Design**	**Patient characteristics**	**Sample size**	**Condition/outcome**	**Trial duration**
Ahmed et al., 2020 [23]	Aerobic training plus rTMS: 20 Hz using 80%–90% of the resting motor threshold Aerobic training plus TENS: Low‐rate mode, 5 Hz, pulse width of 250 *μ*s	RCT	Age: rTMS group = 50.75 ± 4.04 years, TENS group = 50.8 ± 4.30 years	Men = 11; women = 19	Diabetic neuropathic pain Pain (VAS 0–10)	5 consecutive days
Barbarisi et al., 2010 [24]	Pregabalin plus TENS group: 100 Hz, 125 *μ*s	RCT	Age (mean ± SD): Men = 65 ± 8.6 years; women = 64 ± 8.2 years. Pain duration: Men = 15.6 ± 8.8 months; women = 14.9 ± 8.6 months	Men = 15, women = 15 TENS = 16 Sham TENS = 14	Postherpetic neuralgia Pain (VAS 0–100)	4 weeks
Barbarisi et al., 2010 [24]	Pregabalin plus sham TENS group: 100 Hz, 125 *μ*s, no current passed through electrodes	RCT	Age (mean ± SD): Men = 65 ± 8.6 years; women = 64 ± 8.2 years Pain duration: Men = 15.6 ± 8.8 months, women = 14.9 ± 8.6 months	Men = 15, women = 15 TENS = 16 Sham TENS = 14	Postherpetic neuralgia Pain (VAS 0–100)	4 weeks
Bi et al., 2015 [25]	TENS group: 2 Hz, 200 ms Sham TENS group: 2 Hz, 200 ms, no current passed through electrodes	RCT	Age: TENS = 35.5 ± 9 years Sham TENS = 33.6 ± 8.5 years Pain duration: TENS = 7.0 ± 4.1 months, sham TENS: 6.8 ± 3.1 months	Men = 32, women = 16 TENS = 24 Sham TENS = 24	Spinal cord injury Pain (VAS 0–10)	12 weeks
Buchmuller et al., 2012 [26]	TENS mixed group: 80–100 alternated with 2 Hz, 200 ms Sham TENS group: 80–100 alternated with 2 Hz, 200 ms, no current passed through electrodes	RCT	Age: TENS group = 52.0 ± 13 years, sham TENS group = 53.4 ± 12.9 years	TENS group: 71 Sham TENS group: 68	Low back pain Pain (VAS 0–100)	12 weeks
Casale et al., 2013 [27]	TENS group: 100 Hz, 80 ms Laser group: 250 J/cm^2^, 25 W, probe size 1 cm^2^	RCT	Age: TENS group: 56.8 ± 12 years, laser group: 57.3 ± 12.9 years	TENS group = 10 Laser group = 10	Carpal tunnel syndrome Pain (VAS 0–10)	3 weeks
Celik et al., 2013 [28]	TENS group: 4 Hz, 200 *μ*s, intensity: 50 mA Sham TENS 4 Hz, 200 *μ*s, intensity: 50 mA, no current passed through electrodes	RCT	Age: TENS group: 38.18 ± 9.86 years Sham TENS group: 34.81 ± 10.91 years Average pain duration = 19.10 months (1–170 months)	TENS = 17 Men = 4, women = 13 Sham TENS = 16 Men = 11, women = 5	Spinal cord injury Pain (VAS 0–10)	10 days
Eid et al., 2021 [29]	TENS group: 100 Hz, 200 ms Sham TENS: 100 Hz, 200 ms	RCT	Age TENS group: 43.85 ± 5.03; average pain duration: 2.68 ± 0.72 Sham TENS group: 42.22 ± 4.91; average pain duration: 2.51 ± 0.74	TENS group = 26 Sham TENS = 26	Pudendal neuralgia Pain (VAS 0–10)	12 weeks
Eid et al., 2022 [30]	TENS + CMI group: 100 Hz, 200 ms PEMFT + CMI group: 50 Hz	RCT	Age TENS + CMI group = 32.92 ± 5.31 Average pain duration = 3 months Age PEMFT + CMI group = 33.1 ± 4.28	TENS + CMT = 28 PEMFT + CMT = 28	Sciatica Pain (VAS 0–10)	8 weeks
Ghoname et al., 1999 [31]	TENS treatment: 4 Hz, 100 ms PENS treatment: 4 Hz, 100 ms	RCT	Age = 43 ± 19 years Average pain duration = 21 ± 9 months (6–28 months)	Men = 30 Women = 34	Sciatica Pain (VAS 0–10)	3 weeks
Ghoname et al., 1999 [31]	TENS treatment: 4 Hz, 100 ms Sham PENS: 4 Hz, 100 ms, no current passed through electrode	RCT	Age = 43 ± 19 years Average pain duration = 21 ± 9 months (6–28 months)	Men = 30 Women = 34	Sciatica Pain (VAS 0–10)	3 weeks
Gossrau et al., 2011 [32]	TENS: 2 Hz, 30–40 *μ*A Sham TENS	RCT	Age: TENS group: 67.91 ± 12.13 years, sham TENS group: 65.95 ± 7.05 years Pain duration: TENS group: 46.31 ± 51.97 (months); sham TENS: 58.12 ± 60.47	TENS group = 21 participants Sham TENS = 19 participants	Diabetic neuropathic pain Pain (neuropathic pain scale 0–100)	4 weeks
Hoque et al., 2022 [33]	NSAIDs + ADL + TENS (low frequency of 0.5–10 Hz and high intensity of 15–50 mA) NSAIDs + ADL	RCT	Age 21–65 Pain duration: > 3 months	NSAIDs + ADL + TENS group = 40 NSAIDs + ADL group = 40	LBP Pain (VAS 0–10)	8 weeks
Kiliç et al., 2020 [34]	TENS: 100 Hz, 100 ms S‐guided injection: Local anesthetic and steroid	RCT	Age: TENS: 57.25 ± 11.17; US‐guided injection: 51.23 ± 12.58 Pain duration (months): TENS = 19.37 ± 17.82; US‐guided injection = 12.76 ± 13.98	TENS group: 16 participants Injection group = 17 participants	LFCN entrapment Pain (VAS 0–10)	2 weeks
Kiliç et al., 2020 [34]	TENS: 100 Hz, 100 ms Sham TENS	RCT	Age: TENS: 57.25 ± 11.17, sham TENS: 52.76 ± 12.01 Pain duration (months): TENS = 19.37 ± 17.82; sham TENS = 15.28 ± 25.99	TENS group: 16 participants Sham TENS: 21 participants	LFCN entrapment Pain (VAS 0–10)	2 weeks
Kılınç et al., 2014 [35]	TENS 80 Hz, 350 *μ*s, 60 mA	Pre–post	Age: PNP group = 51.75 ± 18.23 years CNP group = 48.5 ± 18.74 years	PNP group = 20 CNP group = 20	Neuropathic pain Pain (VAS 0–10)	4 weeks
Katirci et al., 2023 [36]	TENS: 2 Hz, 200 *μ*s IFC: 80 Hz	RCT	Age TENS = 40.4 ± 10.88 Age IFC = 39.88 ± 7.04	TENS group = 16 IFC = 15	MS Pain (VAS 0–10)	4 weeks
Koca et al., 2014 [37]	TENS 100 Hz, 80 ms Splint therapy	RCT	Age: TENS group = 34.2 ± 5.2 years Splint group = 35.4 ± 4.2 years	TENS group = 20 Women = 13, men = 7 Splint group = 22 Women = 15, men = 7	Carpal tunnel syndrome Pain (VAS 0–10)	3 weeks
Koca et al., 2014 [37]	TENS 100 Hz, 80 ms Interferential current group: 4000 Hz with base 20 Hz	RCT	Age: TENS group = 34.2 ± 5.2 years IFT group = 34.9 ± 4.8 years	TENS group = 20 Women = 13, men = 7 IFT group = 21 Women = 15, men = 6	Carpal tunnel syndrome Pain (VAS 0–10)	3 weeks
Nabi, 2015 [38]	TENS group: 80 Hz, 200 *μ*s Pulse radio frequency sympathectomy delivered as one‐off invasive intervention	RCT	TENS group: 56.63 ± 5.86 years Pulse radio frequency sympathectomy group: 56.76 ± 6.94 years	Women = 29 Men = 31 TENS = 30 Radio frequency = 30	Diabetic neuropathy Pain (NRS 0–10)	12 weeks
Nabi et al., 2021 [39]	Duloxetine: 20 mg/day (1st week), 40 mg/day (2nd week), and 60 mg/day for Weeks 3–12 TENS: 80 Hz, 50 mA	RCT	Duloxetine: 57.16 ± 7.98 years TENS: 58.26 ± 6.93 years Pain duration (months): Duloxetine = 23.56 ± 9.56; TENS = 21.36 ± 9.51	Duloxetine: 16 women and 14 men TENS: 13 women and 17 men	Diabetic neuropathic pain Pain (NRS)	12 weeks
Özkul et al., 2015 [40]	TENS group: 80 Hz, 180 *μ*s VI treatment: 20 min of VI treadmill walking	RCT	Age = 32.33 ± 12.97 years	Men = 18 Women = 6	Spinal cordy injury Pain (VAS 0–10)	2 weeks
Prabhakar et al., 2011 [41]	Joint mobilization TENS 100 Hz, 50 *μ*s Isometric neck exercises	RCT	Joint mobilization = 36.33 ± 9.4 years, TENS = 37.25 ± 9 years, exercise = 39.33 ± 8.6 years	75 participants	Cervical radiculopathy Pain (VAS 0–10)	6 weeks
Reichstein et al., 2005 [42]	TENS: 180 Hz, pulse widths of 4 ms, ≤ 35 mA High‐frequency muscle stimulation: Frequency of 4096–32,768 Hz, pulse widths of ≤ 350 mA	RCT	TENS group: 57.8 ± 12.5 High‐frequency group: 64.2 ± 12.7	TENS group: 11 women and 10 men High‐frequency group: 8 women and 12 men	Diabetic neuropathy Pain (neuropathic pain scale)	3 days
Serry et al., 2015 [43]	TENS group: 15 Hz, 250 *μ*s Exercise group: Aerobic exercise on stationary bicycle	Parallel group design	TENS group: 51.6 ± 4.75 years Exercise group: 51.7 ± 4.44 years	TENS group: *n* = 20 Men = 12, women = 8 Exercise group: *n* = 20 Men = 10, women = 10	Diabetic neuropathy Pain (VAS 0–10)	8 weeks
Serry et al., 2015 [43]	TENS group: 15 Hz, 250 *μ*s Pharmacological group: Regular therapy	Parallel group design	TENS group: 51.6 ± 4.75 years Pharmacological group: 51.95 ± 4.38	TENS group: *n* = 20 Men = 12, women = 8 Pharmacological group: *n* = 20 Men = 10, women = 10	Diabetic neuropathy Pain (VAS 0–10)	8 weeks
Tilak et al., 2016 [44]	TENS group: No TENS frequency details given Mirror group: Intact limb movements performed using mirror	RCT	TENS group: 36.38 ± 9.55 years Mirror group: 42.62 ± 10.69 years	TENS group: Men = 11, women = 2 Mirror group: Men = 12, female = 1	Phantom limb pain Pain (VAS 0–10)	4 days
Cozac et al., 2014 [45]	TENS group: 4 Hz, 200 ms Sham TENS group: 4 Hz, 200 ms, no current passed through electrodes	RCT	TENS group: 31.72 ± 7.7 years Sham TENS group: 28.9 ± 6.1 years	TENS group: Men = 10, woman = 1 Sham TENS: Men = 9, woman = 1	Spinal cord injury Pain (VAS 0–10)	10 days
Zeb et al., 2018 [46]	TENS 80 Hz	Quasiexperimental	Age: 20–60 years	60	Neuropathic pain spinal cord injury (VAS)	8 weeks

Abbreviations: ADL, activities of daily living; CMI, carbamazepine intake; CNP, central neuropathic pain; HZ, hertz; IFC, interferential current; ms, microsecond; MS, multiple sclerosis; NSAIDs, nonsteroidal anti‐inflammatory drugs; PEMFT, pulsed electromagnetic field therapy; PENS, percutaneous electrical nerve stimulation; PNP, peripheral neuropathic pain; RCT, randomized controlled trial; rTMS, repetitive transcranial magnetic stimulation; SD, standard deviation; TENS, transcutaneous electrical nerve stimulation; VAS, visual analogue scale.

**Figure 1:**
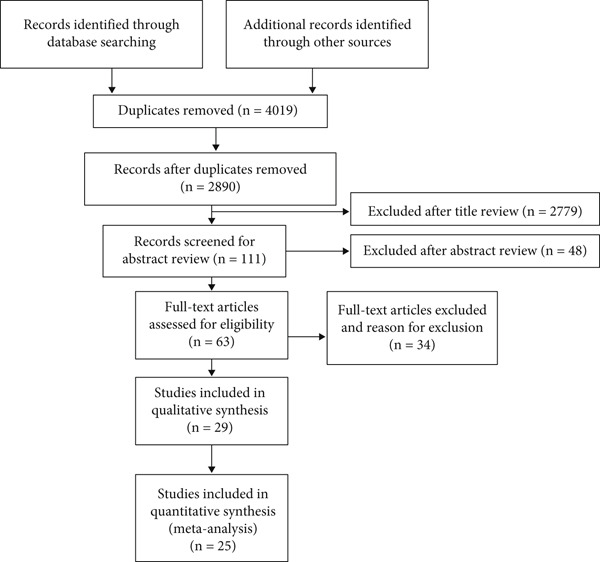
PRISMA flow diagram.

The PRISMA diagram illustrates the selection process (Figure 1). Diabetic neuropathic pain was the most investigated condition [23, 32, 38, 39, 42, 43], followed by neuropathic pain associated with spinal cord injury [25, 28, 40, 46] and carpal tunnel syndrome [27, 37]. Seven studies compared the effectiveness of TENS with sham TENS [24–26, 28, 31, 32, 45], seven studies compared TENS with other forms of electrotherapy [23, 27, 34, 37–39, 42], and six studies compared TENS with other interventions [35, 40, 41, 43, 44, 46].

### 3.2. Effectiveness of TENS Compared to Other Interventions

**Figure 2:**
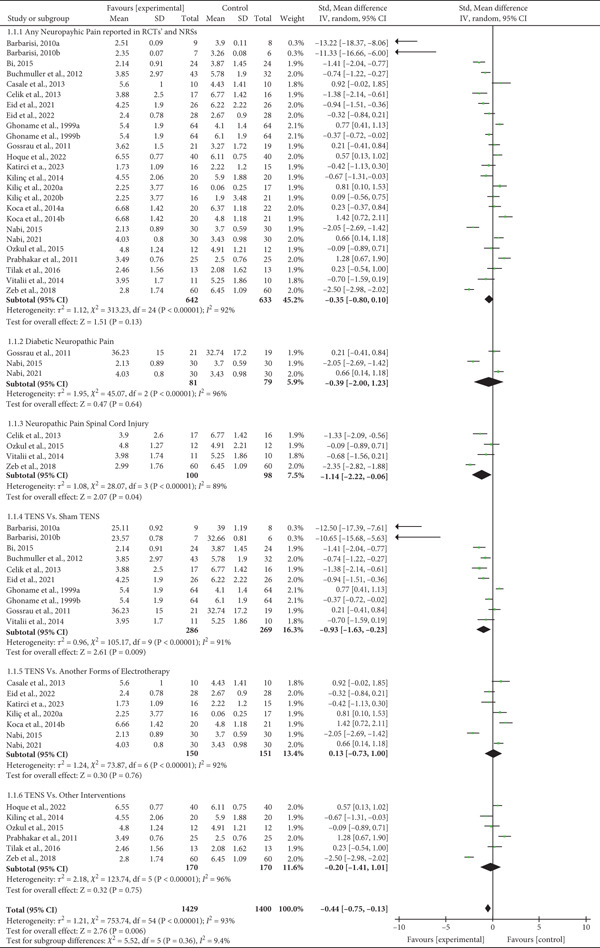
Forest plot showing the effectiveness of TENS for neuropathic pain in general and subgroups by health condition and comparator (RCT = randomized controlled trial, NRS = nonrandomized study, SMD = standardized mean difference, CI = confidence interval).

Twenty‐one studies that provided 25 comparisons of the effectiveness of TENS with different forms of interventions were pooled in a meta‐analysis. There is high‐quality evidence that TENS provides a slight but not clinically significant reduction in neuropathic pain in the short term when compared with placebo SMD = −0.35 (95% CI: −0.80 to 0.10, *p* = 0.13) (Figure 2). In addition, TENS was not significantly different from other forms of electrotherapy SMD = 0.13 (95% CI: −0.73 to 1.00; *p* = 0.76) or other types of interventions SMD = −0.20 (95% CI: −1.41 to 1.01, *p* = 0.75) to reduce pain in the short term.

### 3.3. Effectiveness of TENS for Neuropathic Pain

Results of three studies investigating the effectiveness of TENS for diabetic neuropathic pain [32, 38, 39] showed that there is low‐quality evidence that TENS is not superior to a placebo or other forms of electrotherapy to reduce pain in the short term for patients with diabetic neuropathic pain SMD = −0.39 (95% CI −2.00 to 1.23, *p* = 0.64).

Three studies [4, 40, 46] investigating the use of TENS for neuropathic pain due to spinal cord injury provided moderate‐quality evidence that TENS has a clinically significant effect on pain reduction in these patients SMD = −1.14 (95% CI −2.22 to −0.06, *p* = 0.04) when compared to placebo or other interventions.

The use of TENS for carpal tunnel syndrome was investigated in two studies [27, 37] with moderate risk of bias, providing very‐low quality evidence that TENS is not more effective than laser (SMD = 0.91, 95% CI −0.002 to 1.84, *p* = 0.05) or than the use of splint (SMD = 0.23, 95% CI −0.37 to 0.84). However, TENS was inferior to interferential currency in reducing pain (MD = 1.42, 95% CI 0.72–2.11).

Results from individual studies showed very low‐quality evidence that TENS is more effective than placebo in reducing pain in patients with postherpetic neuralgia [24]^5^, low back pain [26], and meralgia paresthetica [34]. A single study investigated the use of TENS compared to mirror therapy, providing very low‐quality evidence that both interventions have a similar effect on patients with phantom limb pain [44]. Based on very‐low‐quality evidence, TENS is less effective than ultrasound‐guided injection (i.e., corticoid plus anesthetic) and cervical mobilization in patients with meralgia paresthetica [34] and cervical radiculopathy [41], respectively.

### 3.4. Risk of Bias

The risk of bias in the included studies was moderate (mean = 5.72 ± 1.74, range 3–10) on the 10‐point PEDro scale. Table 2 presents the individual risk of bias for each study. Only two studies reported blinding of the therapist(s) [26, 28], three studies stated that assessors were blinded [28, 32, 38], four studies blinded the participants [26, 28, 32, 34], and 11 studies used concealed allocation [26, 28, 31, 32, 34, 36, 40, 44].

**Table 2:** PEDro scale for the risk of bias.

**Studies**	**Eligibility criteria**	**Random allocation**	**Concealed allocation**	**Baseline comparability**	**Blind subjects**	**Blind therapists**	**Blind assessors**	**Adequate follow-up**	**Intention to treat analysis**	**Between-group analysis**	**Point estimates and variability**	**Total**
Ahmed et al., 2020 [23]	1	1	0	0	0	0	0	0	0	1	1	3
Barbarisi et al., 2010 [24]	1	1	0	1	0	0	0	0	1	1	0	4
Barbarisi et al., 2010 [24]	1	1	0	1	0	0	0	0	1	1	0	4
Bi et al., 2015 [25]	1	1	0	1	0	0	0	1	1	1	1	6
Buchmuller et al., 2012 [26]	1	1	1	1	1	1	1	1	1	1	1	10
Casale et al., 2013 [27]	1	1	0	1	0	0	0	1	1	1	1	6
Celik et al., 2013 [28]	1	1	1	0	1	1	1	0	1	1	1	8
Eid et al., 2021 [29]	0	1	0	1	1	0	1	1	0	1	1	7
Eid et al., 2022 [30]	1	1	1	0	1	1	1	0	1	1	1	8
Ghoname et al., 1999 [31]	1	1	1	1	0	0	0	0	1	1	1	6
Ghoname et al., 1999 [31]	1	1	1	1	0	0	0	0	1	1	1	6
Gossrau et al., 2011 [32]	0	1	0	1	1	0	1	1	0	1	1	7
Hoque et al., 2022 [33]	1	1	1	0	1	1	1	0	1	1	1	8
Katirci et al., 2023 [36]	1	1	1	0	1	1	1	0	1	1	1	8
Kiliç et al., 2020 [34]	1	1	1	1	1	0	0	1	0	1	1	7
Kiliç et al., 2020 [34]	1	1	1	1	1	0	0	1	0	1	1	7
Kılınç et al., 2014 [35]	1	1	0	0	0	0	1	0	1	1	1	6
Koca et al., 2014 [37]	1	1	0	1	0	0	0	1	1	1	1	6
Koca et al., 2014 [37]	1	1	0	1	0	0	0	1	1	1	1	6
Nabi et al., 2015 [38]	1	1	0	1	0	0	0	1	0	1	0	4
Nabi et al., 2021 [39]	1	1	0	1	0	0	0	1	0	1	1	5
Özkul et al., 2015 [40]	1	1	1	0	0	0	0	1	1	1	1	6
Prabhakar et al., 2011 [41]	1	1	0	0	0	0	0	1	1	1	1	5
Reichstein et al., 2005 [42]	0	1	0	1	0	0	0	1	0	1	1	5
Serry et al., 2015 [43]	1	1	0	0	0	0	0	0	1	0	1	3
Tilak et al., 2016 [44]	1	1	1	1	0	0	0	1	1	1	1	7
Zeb et al., 2018 [46]	1	1	0	0	1	0	1	0	1	0	1	6

## 4. Discussion

This systematic review and meta‐analysis was aimed at providing recent evidence on the short‐term effect of TENS in reducing pain in patients with neuropathic pain. It included 30 studies investigating TENS use in patients with neuropathic pain due to several health conditions. The existing evidence on the use of TENS ranges from very low to moderate quality.

Our findings demonstrated that TENS provides a slight but not clinically significant reduction in pain in the short term compared to a placebo. The high heterogeneity and risk of bias preclude firm conclusions supporting or refuting the use of TENS for neuropathic pain. However, the main findings of TENS rely on very low‐to‐moderate‐quality evidence that suggests no clinically significant effect when compared to any intervention (i.e., placebo, other forms of electrotherapy, or other therapies). Similar results were found in two recent systematic reviews that investigated the use of TENS for fibromyalgia and chronic neck pain [47, 48]. Johnson et al. concluded that the lack of high‐quality evidence prevents any recommendation in favor of or against TENS in adults with fibromyalgia [47]. In addition, Martimbianco et al. reported very low certainty regarding the effectiveness of TENS compared to placebo (i.e., sham TENS) in individuals with chronic neck pain [48]. Furthermore, a review of eight Cochrane systematic reviews investigating the use of TENS for chronic pain could not reach any recommendation, mainly because of methodological limitations, high heterogeneity, and imprecision of the studies included in these reviews [49].

A pooled analysis showed that TENS provides a similar reduction in pain intensity compared to placebo or other forms of electrotherapies in patients with diabetic neuropathic pain. In contrast, TENS showed a significant effect compared to placebo and other interventions in reducing pain in patients with neuropathic pain associated with spinal cord injury. Our results partially agree with those of a recent systematic review that summarized data from 381 RCTs that investigated the efficacy of TENS for acute and chronic pain in adults [49]. In this review, the authors found a small effect of TENS for reducing pain compared to placebo (SMD = −0.96, 95% CI −1.14 to −0.78) or other treatments (SMD = −0.72, 95% CI −0.95 to −0.50) based on moderate and low certainty evidence, respectively [50]. TENS pain reduction is enhanced by stimulating A*β*, which sends impulses to the dorsal horn of the spinal cord. This activation of interneuron cells in gelatinous substances can increase control of presynapse and close gates for transmission. As a result, the afferent pathway produced by nerve fibers A*δ* is blocked. This means that pain messages carried by these nerve fibers are not transmitted and do not reach the sensory center, leading to reduced pain felt by the patient [51, 52].

The low methodological quality and high heterogeneity between studies, mainly in the type of TENS current and parameters, may influence the results obtained with the application of TENS^51^. The low quality of the trials is a common finding in the reviews summarizing the evidence of TENS [15, 36, 47, 49].

### 4.1. Implications

Pain mechanisms are multifaceted, often causing uncertainty in finite diagnoses. Furthermore, contemporary pain science suggests that pain protects tissue integrity rather than monitoring tissue damage status. Our findings suggest that TENS may benefit pain irrespective of pain characteristics or medical diagnosis, supporting the view that TENS should primarily be indicated according to symptoms, that is, the presence of pain rather than a medical diagnosis. We encourage physiotherapists to consider this evidence when evaluating TENS in the future. Nevertheless, we do not claim that TENS is efficacious for all types of pain because, in this review, TENS provided a slight, not clinically significant reduction in neuropathic pain compared to placebo. For patients with diabetic neuropathic pain, the effect of TENS was similar to placebo and other electrotherapies. However, TENS was better than placebo and other interventions to reduce neuropathic pain in spinal cord injury patients. Hence, there needed to be more studies to judge every diagnosis or pain characteristic.

## 5. Conclusion

TENS generally seems to provide only a small reduction in neuropathic pain compared with a placebo, other electrotherapies, or other interventions. In addition, when investigating multiple studies for specific conditions such as diabetic neuropathic pain and neuropathic pain related to spinal cord injury, the use of TENS could slightly reduce pain only in individuals presenting with the last condition. However, the evidence regarding the effectiveness of TENS for neuropathic pain is poor, and well‐designed studies with adequate sample sizes are needed to develop firm recommendations to support or refute the use of TENS in these patients.

## Conflicts of Interest

The authors declare no conflicts of interest.

## Funding

No funding was received for this manuscript.

## Supporting information


**Supporting Information File S1:** Additional supporting information can be found online in the Supporting Information section. The completed PRISMA 2020 checklist details compliance with reporting guidelines for systematic reviews, including itemized responses to each PRISMA criterion.

## Data Availability

All data generated or analyzed during this study are included in this article and its supporting information files.

## References

[1] Mills S. E. E. , Nicolson K. P. , and Smith B. H. , Chronic Pain: A Review of Its Epidemiology and Associated Factors in Population-Based Studies, British Journal of Anaesthesia. (2019) 123, no. 2, e273–e283, 10.1016/j.bja.2019.03.023, 2-s2.0-85065440894, 31079836. 31079836 PMC6676152

[2] Jensen T. S. , Baron R. , Haanpää M. , Kalso E. , Loeser J. D. , Rice A. S. C. , and Treede R. D. , A New Definition of Neuropathic Pain, 2011, 152, no. 10, 2204–2205, 10.1016/j.pain.2011.06.017, 2-s2.0-80053180821. 21764514

[3] Finnerup N. B. , Haroutounian S. , Kamerman P. , Baron R. , Bennett D. L. H. , Bouhassira D. , Cruccu G. , Freeman R. , Hansson P. , Nurmikko T. , Raja S. N. , Rice A. S. C. , Serra J. , Smith B. H. , Treede R. D. , and Jensen T. S. , Neuropathic Pain: An Updated Grading System for Research and Clinical Practice, Pain. (2016) 157, no. 8, 1599–1606, 10.1097/j.pain.0000000000000492, 2-s2.0-84979716007, 27115670. 27115670 PMC4949003

[4] Baron R. , Binder A. , and Wasner G. , Neuropathic Pain: Diagnosis, Pathophysiological Mechanisms, and Treatment, Lancet Neurology. (2010) 9, no. 8, 807–819, 10.1016/S1474-4422(10)70143-5, 2-s2.0-77955326429. 20650402

[5] Colloca L. , Ludman T. , Bouhassira D. , Baron R. , Dickenson A. H. , Yarnitsky D. , Freeman R. , Truini A. , Attal N. , Finnerup N. B. , Eccleston C. , Kalso E. , Bennett D. L. , Dworkin R. H. , and Raja S. N. , Neuropathic Pain, Nature Reviews Disease Primers. (2017) 3, no. 1, 1–19, 10.1038/nrdp.2017.2, 2-s2.0-85013188047, 28205574. PMC5371025 28205574

[6] Hicks C. W. and Selvin E. , Epidemiology of Peripheral Neuropathy and Lower Extremity Disease in Diabetes, Current Diabetes Reports. (2019) 19, no. 10, 86–86, 10.1007/s11892-019-1212-8, 2-s2.0-85071462578, 31456118. 31456118 PMC6755905

[7] Meacham K. , Shepherd A. , Mohapatra D. P. , and Haroutounian S. , Neuropathic Pain: Central vs. Peripheral Mechanisms, Current Pain and Headache Reports. (2017) 21, no. 6, 10.1007/s11916-017-0629-5, 2-s2.0-85018563975, 28432601. 28432601

[8] Van Hecke O. , Austin S. K. , Khan R. A. , Smith B. H. , and Torrance N. , Neuropathic Pain in the General Population: A Systematic Review of Epidemiological Studies, Pain®. (2014) 155, no. 4, 654–662, 10.1016/j.pain.2013.11.013, 2-s2.0-84896396718, 24291734. 24291734

[9] Bouhassira D. , Lantéri-Minet M. , Attal N. , Laurent B. , and Touboul C. , Prevalence of Chronic Pain With Neuropathic Characteristics in the General Population, Pain. (2008) 136, no. 3, 380–387, 10.1016/j.pain.2007.08.013, 2-s2.0-43549106321. 17888574

[10] GBD 2015 Mortality and Causes of Death Collaborators , Global, Regional, and National Life Expectancy, All-Cause Mortality, and Cause-Specific Mortality for 249 Causes of Death, 1980–2015: A Systematic Analysis for the Global Burden of Disease Study 2015, Lancet. (2016) 388, no. 10053, 1459–1544, 10.1016/S0140-6736(16)31012-1, 2-s2.0-84994158650, 27733281. 27733281 PMC5388903

[11] Cavalli E. , Mammana S. , Nicoletti F. , Bramanti P. , and Mazzon E. , The Neuropathic Pain: An Overview of the Current Treatment and Future Therapeutic Approaches, International Journal of Immunopathology and Pharmacology. (2019) 33, 2058738419838383, 10.1177/2058738419838383, 2-s2.0-85063661591, 30900486. 30900486 PMC6431761

[12] Guastella V. , Mick G. , and Laurent B. , Non Pharmacologic Treatment of Neuropathic Pain, Presse Médicale. (2008) 37, 2 Pt 2, 354–357, 10.1016/j.lpm.2007.11.008, 2-s2.0-41949138554. 18191370

[13] Liampas A. , Rekatsina M. , Vadalouca A. , Paladini A. , Varrassi G. , and Zis P. , Non-Pharmacological Management of Painful Peripheral Neuropathies: A Systematic Review, Advances in Therapy. (2020) 37, no. 10, 4096–4106, 10.1007/s12325-020-01462-3, 32809209. 32809209

[14] Johnson M. I. and Bjordal J. M. , Transcutaneous Electrical Nerve Stimulation for the Management of Painful Conditions: Focus on Neuropathic Pain, Expert Review Neurotherapeutics. (2011) 11, no. 5, 735–753, 10.1586/ern.11.48, 2-s2.0-79955658337, 21539490. 21539490

[15] Gibson W. , Wand B. M. , and O′Connell N. E. , Transcutaneous Electrical Nerve Stimulation (TENS) for Neuropathic Pain in Adults, Cochrane Database Systematic Reviews. (2017) 9, no. 9, Cd011976, 10.1002/14651858.CD011976.pub2, 2-s2.0-85029441432, 28905362. PMC6426434 28905362

[16] Page M. J. , McKenzie J. E. , Bossuyt P. M. , Boutron I. , Hoffmann T. C. , Mulrow C. D. , Shamseer L. , Tetzlaff J. M. , Akl E. A. , Brennan S. E. , Chou R. , Glanville J. , Grimshaw J. M. , Hróbjartsson A. , Lalu M. M. , Li T. , Loder E. W. , Mayo-Wilson E. , McDonald S. , McGuinness L. , Stewart L. A. , Thomas J. , Tricco A. C. , Welch V. A. , Whiting P. , and Moher D. , The PRISMA 2020 Statement: An Updated Guideline for Reporting Systematic Reviews, BMJ. (2021) 372, n71, 10.1136/bmj.n71, 33782057. 33782057 PMC8005924

[17] Riva J. J. , Malik K. M. P. , Burnie S. J. , Endicott A. R. , and Busse J. W. , What is Your Research Question? An Introduction to the PICOT Format for Clinicians, Journal of the Canadian Chiropractic Association. (2012) 56, no. 3, 167–171. 22997465 PMC3430448

[18] De-Morton N. A. , The PEDro Scale is a Valid Measure of the Methodological Quality of Clinical Trials: A Demographic Study, Australian Journal of Physiotherapy. (2009) 55, no. 2, 129–133, 10.1016/S0004-9514(09)70043-1, 2-s2.0-66749148328, 19463084. 19463084

[19] Higgins J. Cochrane Handbook for Systematic Reviews of Interventions. Version 5.1. 0 [updated March 2011]. The Cochrane Collaboration. https://www.cochrane-handbook.org, 2011.

[20] Guyatt G. H. , Oxman A. D. , Kunz R. , Brozek J. , Alonso-Coello P. , Rind D. , Devereaux P. J. , Montori V. M. , Freyschuss B. , Vist G. , Jaeschke R. , Williams J. W.Jr., Murad M. H. , Sinclair D. , Falck-Ytter Y. , Meerpohl J. , Whittington C. , Thorlund K. , Andrews J. , and Schünemann H. J. , GRADE Guidelines 6. Rating the Quality of Evidence--Imprecision, Journal of Clinical Epedemiology. (2011) 64, no. 12, 1283–1293, 10.1016/j.jclinepi.2011.01.012, 2-s2.0-80054997769. 21839614

[21] Olsen M. F. , Bjerre E. , Hansen M. D. , Tendal B. , Hilden J. , and Hróbjartsson A. , Minimum Clinically Important Differences in Chronic Pain Vary Considerably by Baseline Pain and Methodological Factors: Systematic Review of Empirical Studies, Journal of Clinical Epidemiolology. (2018) 101, 87–106.e2, 10.1016/j.jclinepi.2018.05.007, 2-s2.0-85048525770, 29793007. 29793007

[22] Sterne J. A. C. , Sutton A. J. , Ioannidis J. P. A. , Terrin N. , Jones D. R. , Lau J. , Carpenter J. , Rücker G. , Harbord R. M. , Schmid C. H. et al., Recommendations for Examining and Interpreting Funnel Plot Asymmetry in Meta-Analyses of Randomised Controlled Trials, BMJ. (2011) 343, d4002, 10.1136/bmj.d4002, 2-s2.0-79961238388, 21784880. 21784880

[23] Ahmed G. M. , Maher E. A. , Elnassag B. A. E. M. R. , Sayed H. M. , and Kabbash S. I. , Effects of Repetitive Transcranial Magnetic Stimulation Versus Transcutaneous Electrical Nerve Stimulation to Decrease Diabetic Neuropathic Pain, International Journal of Therapy & Rehabilitation. (2020) 27, no. 2, 1–10, 10.12968/ijtr.2018.0037.

[24] Barbarisi M. , Pace M. C. , Passavanti M. B. , Maisto M. , Mazzariello L. , Pota V. , and Aurilio C. , Pregabalin and Transcutaneous Electrical Nerve Stimulation for Postherpetic Neuralgia Treatment, Clinical Journal of Pain. (2010) 26, no. 7, 567–572, 10.1097/AJP.0b013e3181dda1ac, 2-s2.0-77955983101, 20639738. 20639738

[25] Bi X. , Lv H. , Chen B.-L. , Li X. , and Wang X.-Q. , Effects of Transcutaneous Electrical Nerve Stimulation on Pain in Patients With Spinal Cord Injury: A Randomized Controlled Trial, Journal of Physical Therapy Science. (2015) 27, no. 1, 23–25, 10.1589/jpts.27.23, 2-s2.0-84920887510, 25642029. 25642029 PMC4305569

[26] Buchmuller A. , Navez M. , Milletre-Bernardin M. , Pouplin S. , Presles E. , Lantéri-Minet M. , Tardy B. , Laurent B. , Camdessanché J. P. , and Lombotens Trial Group , Value of TENS for Relief of Chronic Low Back Pain With or Without Radicular Pain, Eropean Journal of Pain. (2012) 16, no. 5, 656–665, 10.1002/j.1532-2149.2011.00061.x. 22337531

[27] Casale R. , Damiani C. , Maestri R. , and Wells C. D. , Pain and Electrophysiological Parameters Are Improved by Combined 830-1064 High-Intensity LASER in Symptomatic Carpal Tunnel Syndrome Versus Transcutaneous Electrical Nerve Stimulation. A Randomized Controlled Study, European Journal of Physical and Rehabilitation Medicine. (2013) 49, no. 2, 205–211. 22820819

[28] Celik E. C. , Erhan B. , Gunduz B. , and Lakse E. , The Effect of Low-Frequency TENS in the Treatment of Neuropathic Pain in Patients With Spinal Cord Injury, Spinal Cord. (2013) 51, no. 4, 334–337, 10.1038/sc.2012.159, 2-s2.0-84878307270, 23295472. 23295472

[29] Eid M. M. , Rawash M. F. , Sharaf M. A. , and Eladl H. M. , Effectiveness of Transcutaneous Electrical Nerve Stimulation as an Adjunct to Selected Physical Therapy Exercise Program on Male Patients With Pudendal Neuralgia. A Randomized Controlled Trial, Clinical Rehabilitation. (2021) 35, no. 8, 1142–1150, 10.1177/0269215521995338. 33611923

[30] Eid M. M. , Hamed N. S. , Abdelbasset W. K. , Elkholi S. M. , Eladl H. M. , and Bahey El-Deen H. A. , A Comparative Study Between Transcutaneous Electrical Nerve Stimulation and Pulsed Electromagnetic Field Therapy in the Management of Post-Herpetic Neuralgia of the Sciatic Nerve, Medicine. (2022) 101, no. 44, e31433, 10.1097/MD.0000000000031433, 36343068. 36343068 PMC9646515

[31] Ghoname E. A. , White P. F. , Ahmed H. E. , Hamza M. A. , Craig W. F. , and Noe C. E. , Percutaneous Electrical Nerve Stimulation: An Alternative to TENS in the Management of Sciatica, Pain. (1999) 83, no. 2, 193–199, 10.1016/S0304-3959(99)00097-4, 2-s2.0-0032852186, 10534590. 10534590

[32] Gossrau G. , Wahner M. , Kuschke M. , Konrad B. , Reichmann H. , Wiedemann B. , and Sabatowski R. , Microcurrent Transcutaneous Electric Nerve Stimulation in Painful Diabetic Neuropathy: A Randomized Placebo-Controlled Study, Pain Medicine. (2011) 12, no. 6, 953–960, 10.1111/j.1526-4637.2011.01140.x, 2-s2.0-79959278494, 21627767. 21627767

[33] Hoque D. M. A. , Hossain D. M. S. , Ahmed D. M. , Sadeque D. A. Z. , Haque D. A. , and Ilias M. , Effects of Transcutaneous Electrical Nerve Stimulation in Patients With Chronic Non-Specific Low Back Pain, Scholars Journal of Applied Medical Sciences. (2022) 10, no. 7, 1094–1104, 10.36347/sjams.2022.v10i07.010.

[34] Kiliç S. , Özkan F. Ü. , Külcü D. G. , Öztürk G. , Akpinar P. , and Aktas I. , Conservative Treatment Versus Ultrasound-Guided Injection in the Management of Meralgia Paresthetica: A Randomized Controlled Trial, Pain Physician. (2020) 23, no. 3, 253–262. 32517391

[35] Kılınç M. , Livanelioğlu A. , Yıldırım S. A. , and Tan E. , Effects of Transcutaneous Electrical Nerve Stimulation in Patients With Peripheral and Central Neuropathic Pain, Journal of Rehabilitation Medicine. (2014) 46, no. 5, 454–460, 10.2340/16501977-1271, 2-s2.0-84902978566. 24549206

[36] Kirmaci Z. İ. , Adigüzel H. , Göğremiş M. , Kirmaci Y. Ş. , İnanç Y. , and Tuncel B. D. , The Effect of Transcutaneous Electrical Nerve Stimulation (TENS) and Interferential Currents (IFC) on Pain, Functional Capacity, and Quality of Life in Patients With Multiple Sclerosis: A Randomized Controlled, Single-Blinded Study, Multiple Sclerosis and Related Disorders. (2023) 71, 104541, 10.1016/j.msard.2023.104541, 36738692. 36738692

[37] Koca I. , Boyaci A. , Tutoglu A. , Ucar M. , and Kocaturk O. , Assessment of the Effectiveness of Interferential Current Therapy and TENS in the Management of Carpal Tunnel Syndrome: A Randomized Controlled Study, Rheumatology International. (2014) 34, no. 12, 1639–1645, 10.1007/s00296-014-3005-3, 2-s2.0-84939879280. 24728028

[38] Nabi B. , Sedighinejad A. , Haghighi M. , Biazar G. , Hashemi M. , Haddadi S. , and Fathi A. , Comparison of Transcutaneous Electrical Nerve Stimulation and Pulsed Radiofrequency Sympathectomy for Treating Painful Diabetic Neuropathy, Anesthesiology Pain Medicine. (2015) 5, no. 5, e29280, 10.5812/aapm.29280, 2-s2.0-84942938495, 26587405. 26587405 PMC4644305

[39] Nabi B. N. , Saberi A. , Eghbali B. B. , Hosseininezhad M. , Biazar G. , Malekabadi A. A. , and Mirmansouri L. , Efficacy and Safety of Tens and Duloxetine in Patients With Painful Diabetic Neuropathy: A Single Blind Randomized Clinical Trial, Journal of Advances in Medical and Biomedical Research. (2021) 29, no. 136, 286–292, 10.30699/jambs.29.136.286.

[40] Özkul Ç. , Kılınç M. , Yıldırım S. A. , Topçuoğlu E. Y. , and Akyüz M. , Effects of Visual Illusion and Transcutaneous Electrical Nerve Stimulation on Neuropathic Pain in Patients With Spinal Cord Injury: A Randomised Controlled Cross-Over Trial, Journal of Back and Musculoskelet Rehabiltation. (2015) 28, no. 4, 709–719, 10.3233/BMR-140573, 2-s2.0-84954467582, 25502348. 25502348

[41] Prabhakar R. and Ramteke G. , Cervical Spinal Mobilization Versus TENS in the Management of Cervical Radiculopathy: A Comparative, Experimental, Randomized Controlled Trial, Indian Journal of Physiotherapy & Occupational Therapy. (2011) 5, 128–133.

[42] Reichstein L. , Labrenz S. , Ziegler D. , and Martin S. , Effective Treatment of Symptomatic Diabetic Polyneuropathy by High-Frequency External Muscle Stimulation, Diabetologia. (2005) 48, no. 5, 824–828, 10.1007/s00125-005-1728-0, 2-s2.0-20044362599, 15830180. 15830180

[43] Serry Z. , Mossa G. , Elhabashy H. , Elsayed S. , Elhadidy R. , Azmy R. , and Mokhtar A. , Transcutaneous Nerve Stimulation Versus Aerobic Exercise in Diabetic Neuropathy, Egyptian Journal of Neurology, Psychiatry and Neurosurgery. (2016) 53, no. 2, 124–129.

[44] Tilak M. , Isaac S. A. , Fletcher J. , Vasanthan L. T. , Subbaiah R. S. , Babu A. , Bhide R. , and Tharion G. , Mirror Therapy and Transcutaneous Electrical Nerve Stimulation for Management of Phantom Limb Pain in Amputees - A Single Blinded Randomized Controlled Trial, Physiotherapy Research International. (2016) 21, no. 2, 109–115, 10.1002/pri.1626, 2-s2.0-84926390687, 25832306. 25832306

[45] Cozac V. and Pascal O. , The Efficiency of Transcutaneous Electrical Nerve Stimulation in Association With Gabapentin in the Treatment of Neuropathic Pain in Patients With Spinal Cord Injury, Romanian Journal of Neurology. (2014) 13, no. 4, 193–196, 10.37897/RJN.2014.4.4.

[46] Zeb A. , Arsh A. , Bahadur S. , and Ilyas S. M. , Effectiveness of Transcutaneous Electrical Nerve Stimulation in Management of Neuropathic Pain in Patients With Post Traumatic Incomplete Spinal Cord Injuries, Pakistan Journal of Medical Sciences. (2018) 34, no. 5, 1177–1180, 10.12669/pjms.345.15659, 2-s2.0-85056640829. 30344571 PMC6191807

[47] Johnson M. I. , Claydon L. S. , Herbison G. P. , Jones G. , and Paley C. A. , Transcutaneous Electrical Nerve Stimulation (TENS) for Fibromyalgia in Adults, Cochrane Database Systematic Review. (2017) 10, no. 10, Cd012172, 10.1002/14651858.CD012172.pub2, 2-s2.0-85030873638, 28990665. PMC6485914 28990665

[48] Martimbianco A. L. C. , Porfírio G. J. , Pacheco R. L. , Torloni M. R. , and Riera R. , Transcutaneous Electrical Nerve Stimulation (TENS) for Chronic Neck *Pain* , Cochrane Database of Systematic Reviews. (2019) 12, no. 12, 10.1002/14651858.CD011927.pub2, 31830313. PMC6953309 31830313

[49] Gibson W. , Wand B. M. , Meads C. , Catley M. J. , and O′Connell N. E. , Transcutaneous Electrical Nerve Stimulation (TENS) for Chronic Pain - an Overview of Cochrane Reviews, Cochrane Database Systemstic Review. (2019) 4, no. 4, Cd011890, 10.1002/14651858.CD011890.pub3, 30941745. PMC6446021 30941745

[50] Johnson M. I. , Paley C. A. , Jones G. , Mulvey M. R. , and Wittkopf P. G. , Efficacy and Safety of Transcutaneous Electrical Nerve Stimulation (TENS) for Acute and Chronic Pain in Adults: A Systematic Review and Meta-Analysis of 381 Studies (the Meta-TENS Study), BMJ Open. (2022) 12, no. 2, e051073, 10.1136/bmjopen-2021-051073, 35144946. PMC8845179 35144946

[51] Lu Y. , Dong H. , Gao Y. , Gong Y. , Ren Y. , Gu N. , Zhou S. , Xia N. , Sun Y. Y. , Ji R. R. , and Xiong L. , A Feed-Forward Spinal Cord Glycinergic Neural Circuit Gates Mechanical Allodynia, Journal of Clinical Investigation. (2013) 123, no. 9, 4050–4062, 10.1172/JCI70026, 2-s2.0-84883515305, 23979158. 23979158 PMC4381282

[52] Peng W. W. , Tang Z. Y. , Zhang F. R. , Li H. , Kong Y. Z. , Iannetti G. D. , and Hu L. , Neurobiological Mechanisms of TENS-Induced Analgesia, NeuroImage. (2019) 195, 396–408, 10.1016/j.neuroimage.2019.03.077, 2-s2.0-85064175411, 30946953. 30946953 PMC6547049

